# Barriers and facilitators of a large clinical trial on prevention of HIV transmission through breastfeeding in Lusaka, Zambia: a qualitative study

**DOI:** 10.1186/s12889-024-20855-5

**Published:** 2024-12-02

**Authors:** Anaïs Mennecier, Beauty Matoka, Maria Melany Wilfred-Tonga, Catherine Chunda-Liyoka, Mwiya Mwiya, Nicolas Nagot, Jean-Pierre Molès, Philippe Van de Perre, Chipepo Kankasa, Rachel King, for the PROMISE-EPI social science sub-study group, Anaïs Mennecier, Beauty Matoka, Maria Melany Wilfred-Tonga, Catherine Chunda-Liyoka, Mwiya Mwiya, Nicolas Nagot, Jean-Pierre Molès, Philippe Van de Perre, Chipepo Kankasa, Rachel King, Morgana d’Ottavi, David Rutagwera, Sylvester Banda, Faith Sitali, Chayson Maunda, Mwape Kelvin Chisala, Richard Kandela, Kennedy Changwa Sikambale, Mwape Chibale, Sara Phiri, Gertrude Munanjalaa, Vera Ndulumina Kawanga, Eric Maseko Phiri, Shanzi Mulenga, Jenala Nyirenda Hapenga, Kapambwe Mulenga

**Affiliations:** 1 Pathogenesis and Control of Chronic and Emerging Infections, Univ Montpellier, INSERM, EFS, Univ Antilles, Montpellier, France; 2 Pediatric Centre of Excellence, University Teaching Hospital, Lusaka, Zambia

**Keywords:** Vertical transmission, MTCT, HIV prevention, Point-of-care, Post-natal prophylaxis, Qualitative, Breast-feeding, Zambia, Africa

## Abstract

**Background:**

PROMISE-EPI trial evaluated a combination of interventions to prevent HIV transmission during breastfeeding. It showed a reduced postnatal transmission compared to the standard of care. The intervention combined identification of infants at high risk of infection using a point of care assay (POC) for early infant diagnosis and monitoring maternal viral load (VL) at 6 weeks and 6 months. A single-drug post-natal prophylaxis (PNP) was immediately initiated for high risk infants (maternal VL ≥ 1000 cp/mL). In Zambia, the national guidelines standard of care differs by 1) using three-drug PNP; 2) quarterly monitoring of maternal VL; 3) maternal VL testing in central labs. We explored the facilitators and barriers of this innovative prevention package to guide future scale-up.

**Methods:**

Qualitative methods were used to gather information on PROMISE-EPI trial delivery, context, and behaviors. PROMISE-EPI intervention and control participants, staff members and health care professionals were interviewed. Verbatim transcripts were coded using a priori and emerging codes. Analysis was conducted using the RE-AIM (Reach, Effectiveness, Adoption, Implementation, Maintenance) framework. The determinants were categorized into the 5 domains of the Consolidated Framework for Implementation Research (CFIR) to better identify the causes of intervention success or failure among the 5 RE-AIM components.

**Results:**

A total of 37 individual interviews and 15 focus group discussions were conducted. Facilitators included the importance of the connection between the key elements of the intervention (POC and PNP) for immediate clinical action. Rapid maternal VL results induce several positive downstream behaviors in mothers and healthcare professionals, including increased trust in health care system. These can be quickly reversed when point of care testing is sub-optimal, as during the COVID-19 pandemic. Furthermore, the secondary elements of the intervention beyond POC and PNP; namely a warm welcome, a dedicated space, detailed and dedicated counselling, reimbursement for transport, solar panels and batteries, reminders and additional staff; were identified as facilitating its acceptability and fidelity.

**Conclusion:**

This study provides new elements to better understand the reduced HIV transmission with the PROMISE-EPI intervention. It also highlights potential gaps between the package proposed in the trial and what can be applied in less controlled, ‘real life’ settings.

**Supplementary Information:**

The online version contains supplementary material available at 10.1186/s12889-024-20855-5.

## Background

Mother to child transmission (MTCT) of HIV is a major public health concern, with currently 130,000 children newly infected with HIV in 2022 [1]. The sharp decline in pediatric infection over the past two decades is mainly due to the significant decline in perinatal transmissions. Breastfeeding remains a critical period, accounting for more than 50% of the current MTCTs [2].

Antiretroviral therapy (ART), leading to maternal viral load suppression, and infant post-natal prophylaxis (PNP) are the two pillars of post-natal prevention of mother to child transmission (PMTCT) program. Nevertheless, regular maternal viral load testing can be a challenge in low-middle income settings hampering sustainable monitoring of ART adherence and efficacy [3]. Furthermore, there is no international consensus on how PNP should be extended beyond 6 to 12 weeks of life for breastfed infants exposed to HIV, or who should benefit from it. Therefore, every country has developed their own recommendations [4, 5].

The PROMISE-EPI trial, implemented between December 2019 and September 2022, aimed to prevent the HIV MTCT by identifying breastfed children at high risk of HIV acquisition beyond the six weeks of age. The prevention package included two point of care (POC) tests for infant HIV diagnosis and for maternal viral load monitoring at 6 weeks and 6 months. Immediate results enabled quick initiation of ART if the infant tested positive, and a single-drug PNP (lamivudine syrup) in infants exposed to HIV in the event of maternal viral load (VL) higher than 1000 cp/mL. This prevention package was evaluated versus standard of care through a randomized clinical trial in Zambia and Burkina Faso [6]. In Zambia, where 89% of participants were recruited, the national guidelines standard of care differed from the intervention by 1) the type of PNP (three drug PNP); 2) quarterly monitoring of maternal VL; 3) maternal VL testing in external centralized laboratory. The PROMISE-EPI prevention package proved efficacious in preventing HIV transmission and was safe [7].

While demonstrating the efficacy of an intervention is a necessary and major step, it is not an end in itself. Without further research, evidence-based practices are, at best, belatedly incorporated into routine [8, 9], and the gap between effective and delivered care limits the usefulness of the new intervention. Implementation science approaches aim to facilitate successful uptake of evidence-based practice [10]. By focusing on contextual inquiry, these approaches provide information on the elements that need to be taken into consideration to limit the evidence-practice gap [10]. We gathered information on the PROMISE-EPI trial delivery, context and behavior through a qualitative sub-study first exploring facilitators and barriers to infant post-natal prophylaxis in Zambia. We found better acceptability of the PNP given in PROMISE-EPI trial compared to the PNP offered by the national program, especially because of its ready-to-use formulation and better palatability [11]. In the current manuscript we aim to explore factors influencing the effectiveness of the PROMISE-EPI prevention package in Zambia using the Reach, Effectiveness, Adoption, Implementation and Maintenance (RE-AIM) and the five domains of the Consolidated Framework for Implementation Research (CFIR) frameworks.

## Methods

### Context and settings

The protocol of PROMISE-EPI trial is described elsewhere [6]. Briefly, the study was proposed to all mothers infected with HIV coming for the second visit of the expanded program on immunization (EPI-2), when their babies were about six weeks old in Zambia, and who met the eligibility criteria. Following the infant HIV diagnosis using a GeneXpert POC, the breastfeeding mother/HIV exposed uninfected infant pairs willing to participate in the PROMISE-EPI trial were randomized to the intervention or the control arm and followed until the baby was one-year-old. All infants, regardless of arm, were re-tested for HIV at 6 months (M6) and 12 months (M12) through a POC GeneXpert test. The differences in care received by participants in the intervention and control arms are detailed in the Table 1. The Zambian national guidelines changed in 2020 and 98% of PROMISE-EPI participants were recruited after the release of the 2020 guidelines [12].

**Table 1:** Difference in care received in the PROMISE-EPI intervention and control arms, Lusaka, Zambia

Intervention arm	Control arm	
PROMISE-EPI prevention package	Zambian standard of care	
- POC for maternal viral load monitoring at EPI-2 and M6 - Immediate single-drug (lamivudine) infant prophylaxis initiation in case of mother viral load ≥ 1000cp/ml - Monthly visits for infants on lamivudine	**Before 2020:** - Viral load monitoring every 6 months during the breastfeeding period	**After 2020:** - Extended three drug infant prophylaxis (AZT/3TC + NVP) in case of maternal viral load ≥ 1000 cp/ml at 10 weeks and maternal viral load follow-up every 3 months

*POC* point-of-care, *EPI-2* second visit of the expanded program on immunization, *AZT* zidovudine, *3TC* lamivudine, *NVP* nevirapine

The clinical trial was implemented in four health facilities in Lusaka. A team specifically hired for the PROMISE-EPI study, consisting of fourteen nurses and clinical officers, cared for the participants in a dedicated physical space in each clinic. They were in charge of all the activities including visit reminders (several phone calls), sample collection, POC testing and results disclosure, counselling, medical care and providing lamivudine. Counseling included giving pre and post HIV test results counselling, how to deal with stigma, how to take care of infants, risk of HIV transmissions, breastfeeding advice, adherence to ART, instruction on lamivudine administration and adherence. PROMISE-EPI staff members were assisted by community health workers from the health facilities to re-contact mothers lost to follow-up, via home visits.

All PROMISE-EPI participants, from both arms, received 100 Kwachas (5 USD) compensation for each PROMISE-EPI trial visit.

### Study design, participants’ selection and sampling

In Zambia, the qualitative sub-study was implemented in all four PROMISE-EPI sites, using individual interviews and focus group discussions (FGDs). These in depth interviews were conducted with three groups of participants: 1) Health care professionals (HCP) contributing to the PMTCT program in the facilities where the trial was implemented, 2) PROMISE-EPI staff members and 3) PROMISE-EPI participants from both study arms. All PROMISE-EPI staff members were interviewed and a purposive sampling approach was applied to select the participants among the HCP and the PROMISE-EPI participants. Out of the 987 PROMISE-EPI participants who consented to be approached for the qualitative sub-study, those with any of the following characteristics were considered at higher risk of HIV transmission and a sample of them was included: 1) mothers who dropped out the PMTCT program at the time of EPI-2 visit (defined as exposed infants who did not have access to HIV diagnosis or PNP at birth), 2) mothers with a viral load ≥ 1000 cp/mL, and 3) mothers still breastfeeding baby at 12 months of age. Written informed consent were also obtained from PROMISE-EPI staff members and HCP contributing to the PMTCT program in the facilities where the trial was implemented.

After reviewing the ten first transcripts, a meeting of the study team determined the approximate number of additional interviews needed to reach content saturation.

Triangulation was obtained through the involvement of the four sites, inclusion of three groups of participants and conducting individual interviews and focus groups discussions, as well as discussion with the study team [13].

### Data collection

The PROMISE-EPI staff members conducted the face to face individual interviews and focus group discussions. PROMISE-EPI staff interviewed each other to gain an understanding of their experience during study implementation. After initial training for the interviewers, further steps were taken to ensure the quality of data collection. A review of the initial transcripts by the social science researcher (RK) provided an opportunity to discuss any difficulties encountered with the study team, at an early stage in the study. The interviewers also benefited from feedback on techniques following the participation of the social science researcher in a number of focus group discussions.

The interviews, lasting about 1 h each, were based on a semi-structured guide. They were conducted between December 2021 and September 2022 in the PROMISE-EPI study rooms. Participants were asked about the factors related to the acceptability, feasibility, adherence and usability of PROMISE-EPI prevention package, how they felt being part of the study and their suggestions to improve these services.

PROMISE-EPI participants received compensation for travel to the study site and for the time spent in interviews (100 Kwachas = 5 USD).

The interviews were conducted in Bemba, Nyanja or English and translated into English, if necessary, during transcription by the interviewer. Interviews were audio-recorded and anonymity was preserved by assigning a number at the beginning of the interview.

### Data analysis

An initial code-book was designed on the basis of codes determined a priori using the interview guide (see Appendix). It was then adapted on the basis of themes emerging in five transcripts initially double-coded by two researchers (RK and AM) and compared to ensure reliability. Any discrepancies between the coding were discussed and resolved. The remaining transcripts were coded by one researcher (AM) using Excel software and reviewed by the second (RK). Only participants’ answers clearly identifying the reference to the standard of care or the PROMISE-EPI intervention were considered in the analysis.

The RE-AIM framework, conceptualized to evaluate the public health significance of an intervention, has been considered a useful tool to qualitatively assess the prevention package proposed in PROMISE-EPI trial [14–16]. The ideas that emerged from the interviews were processed through questions adapted to our context on the basis of the RE-AIM descriptions [14–16]. Nevertheless, the RE-AIM framework is not detailed enough to identify the causes of success or failure of the intervention in each of its five elements [17]. The integration of the five domains of CFIR into to RE-AIM framework fills this gap by providing a structure for examination of factors and assuring completeness of understanding [16, 18–20].

Trustworthiness was enhanced by checking with members and feedback was also obtained from dissemination with national stakeholders, the study team and PROMISE-EPI staff.

PROMISE-EPI participant characteristics were retrieved from PROMISE-EPI electronic Case Report Form (eCRF). Statistical analyses, including Student t test for mother’s age and Fisher exact test for other variables, were carried out using Stata 16.1 (Stata Corp, College Station, Texas). Missing data were omitted from percentage calculations.

## Results

In total, 37 individual interviews and 15 focus groups of discussions were included in this analysis from a total of 122 participants: 81 PROMISE-EPI participants, 13 PROMISE-EPI staff and 28 HCP including nurses, counselors, lab technicians, pharmacists, community health workers, one non-governmental organization (NGO) representative and a representative of the Ministry of Health.

Interviewed participants were not significantly different from non-interviewed PROMISE-EPI participants with the exception of the following variables: study arm; maternal viral load ≥ 1000cp/mL at EPI-2 and M6 and infants eligible for lamivudine PNP (Table 2). Although we interviewed all types of participants, we focused on those most at risk (mothers with at least one viral load ≥ 1000 cp/mL) and those who had received the full PROMISE-EPI prevention package. Furthermore, interviewed mothers were significantly less likely to be lost to follow-up at the M12 PROMISE-EPI visit (1/80, 1.3%) compared to the non-interviewed mothers (270/1262, 21.4%; *p* = < 0.001).

**Table 2:** Comparison of baseline characteristics between PROMISE-EPI participants interviewed and non-interviewed, Lusaka, Zambia

		**PROMISE-EPI participants interviewed**	**PROMISE-EPI participants not interviewed**	***P* value**
		***N* = 80**^**a**^	***N* = 1262**	
Intervention arm		49 (61.3%)	619 (49%)	0.038
Mothers				
Age	mean (sd)	30.0 (6.5)	30.5 (5.9)	0.47
Time of HIV diagnosis	Before this last pregnancy	53 (66.3%)	899/1260 (71.3%)	0.37
	During/after this last pregnancy	27 (33.8%)	361/1260 (28.7%)	
Mothers taking ART		76 (95.0%)	1243 (98.5%)	0.044
Highest level of education	None	5 (6.3%)	82 (6.5%)	0.87
	Primary	29 (36.3%)	494 (39.1%)	
	Secondary or more	46 (57.5%)	686 (54.4%)	
Employment situation during pregnancy	Not working	56 (70.0%)	872 (69.1%)	0.61
	Working (formal sector)	5 (6.3%)	122 (9.7%)	
	Working (informal sector)	17 (21.3%)	248 (19.7%)	
	Studying	2 (2.5%)	20 (1.6%)	
Marital status	Married/cohabitating	63 (78.8%)	1,047 (83.0%)	0.43
	Single	15 (18.8%)	173 (13.7%)	
	Divorced/separate/widowed	2 (2.5%)	42 (3.3%)	
HIV Status known by partner	No	7/71 (9.9%)	88/974 (9.0%)	0.29
	Yes	60/71 (84.5%)	858/974 (88.1%)	
	Not applicable (no partner)	4/71 (5.6%)	28/974 (2.9%)	
Maternal VL > = 1000cp/mL at EPI-2 (both arms)		38 (47.5%)	105/1222 (8.6%)	< 0.001
Maternal VL > = 1000cp/mL at M6		23/76 (30.3%)	64/1054 (6.1%)	< 0.001
Still breastfeeding at M12		63/79 (79.7%)	734/990 (74.1%)	0.35
Infants				
Sex	Female	40 (50.0%)	611 (48.4%)	0.82
Six week PNP at birth (3 drugs)		74 (92.5%)	1217/1262 (96.4%)	0.12
Infant eligible to lamivudine at EPI-2 (intervention arm)		35 (43.8%)	37 (2.9%)	< 0.001
Newly eligible to lamivudine at M6 (mother on the intervention arm newly unsuppressed)		3 (3.8%)	13 (1.0%)	0.065

*ART* Antiretroviral therapy, *VL* Viral load, *PNP* Post-natal-prophylaxis, *EPI-2* second visit of the expanded program on immunization

^a^ The PROMISE-EPI identifier was not recorded for one PROMISE-EPI participant

All infants were between 6 and 18 months old at the time of interviews.

**Fig. 1:**
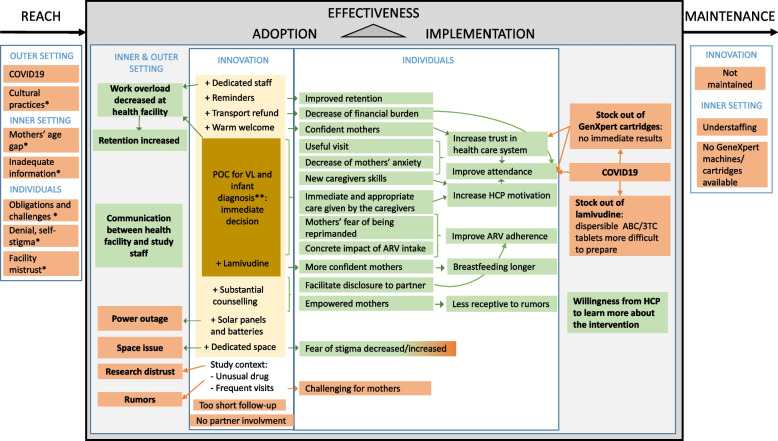
Barriers and facilitators of PROMISE-EPI intervention through RE-AIM and CFIR frameworks. Legend: Facilitators are in green and barriers in orange; the main elements of the PROMISE-EPI package are in dark yellow and the secondary elements are in light yellow; *Not specifically reported for EPI-2, but for MCH attendance; ** POC for early infant diagnosis was offered to the participants of both arms. VL: Viral load; POC: Point of care; HCP: Health care professionals; ABC/3TC: abacavir/lamivudine

The study findings are schematized in Fig. 1 and Appendix.

## Reach

### Is the intervention reaching the target populations?

#### Outer setting

The COVID-19 pandemic was reported to be a barrier to attend the EPI-2 visit, and therefore to participating in the PROMISE-EPI study. Restrictions in facilities, 6-month ART dispensing, inability to afford buying a mask, fear of getting sick, fear of being swabbed or vaccinated for COVID-19 all had a negative impact on attendance at health facilities.

“Most mothers were shunning the facility for fear of getting sick [COVID-19], the attendance of mothers at 6–8 weeks had gone down.” (Counsellor; individual interview).

#### Individuals, outer and inner settings not specifically reported for EPI-2

Other obstacles to attending Maternal and Child Health (MCH) were reported by mothers, without being specifically attributed to the EPI-2 visit. Barriers related to individuals included maternal obligations and challenges such as caring for other children, financial issues and travel for personal reasons or to deliver near family. Not willing to be mixed with younger/older mothers was an inner setting barrier. Other obstacles were identified specifically for mothers living with HIV who were aware of their status: denial, self-stigmatization, cultural practices, inadequate information received and distrust of health care facility were barriers to continuity of care.

## Effectiveness

### What are the impacts of the PROMISE-EPI prevention package on important individual outcomes including behavioral outcomes and quality of life?

#### Individuals

Immediate results obtained through POC for maternal viral load and infant HIV diagnosis had positive impacts on mothers’ anxiety. It increased their trust in the health system and therefore their attendance to follow-up visits. This perception was shared by mothers and HCPs. The latter emphasized that if POC was used routinely, mothers would come, even in cases of financial hardship, because they would be sure to benefit from their visit.

“After seeing the baby result, I gained trust in Promise EPI staff and I would call to find out about my next appointment because I was more interested in the baby’s result.” (Mother 31 yrs; FGD)

POC use for viral load monitoring encourages mothers to take their antiretrovirals (ARVs). On the one hand, a controlled viral load allows them to see a concrete impact of good ARV adherence and can be experienced as a reward. On the other hand, mothers are aware that nurses will know that they are not taking their ARV drugs properly if their viral load is not suppressed and are afraid of being reprimanded.

“I also came to know that if I don’t take drugs, the viral load will be high and they are going to know that I don’t take drugs... because of that, I was forced to take drugs. I was afraid of being scolded.” (Mother 23yrs; FGD)

The PROMISE-EPI prevention package, including immediate infant diagnosis and viral load results, as well as the initiation of lamivudine in case of unsuppressed maternal viral load, resulted in some mothers feeling more confident and breastfeeding longer.

“I also decided to breastfeed my baby beyond 1 year because the baby tested negative for HIV at 6 months and because of the prophylaxis medication which I was getting from Promise – EPI study” (Mother 39 yrs; FGD)

The counselling provided by the PROMISE-EPI staff empowered many mothers. It made them less receptive to the rumors spread by the community, instilled confidence to encourage others and facilitated HIV status disclosure to their partners, which they reported had a positive impact on their ARV adherence.

“The counseling that you have been giving us throughout has made me strong that I can even encourage others.” (Mother 27 yrs; individual interview)

## Adoption

### Are settings and intervention agents willing to initiate the PROMISE-EPI study?

#### Individuals

Dedicated staff were involved in PROMISE-EPI study. The study nurses and clinical officers were in charge of all aspects of the study in the health facilities including the POC testing. They reported enjoying their work because of the benefit to the community in terms of PMTCT and because they learned new skills, particularly how to use the GeneXpert machines.

“Personally, I have learnt a lot, I have learnt how to operate a GeneXpert machine, and just the study itself has been very educative because I have learnt some things I never knew in PMTCT.” (PROMISE-EPI staff; FGD)

The main wish of interviewed HCP for improving PMTCT services is the implementation of POC, used in PROMISE-EPI. The immediate results enabled caregivers to provide appropriate care, which increased their motivation for their work.

#### Inner setting

The HCP in the facilities where the PROMISE-EPI trial was carried out, but who did not have any involvement in the trial, wanted to know more about the details of the intervention and the results of the trial to improve the routine services they provide.

“When we give them appointments from our end, they will not come, but they will go the other end (where PROMISE was implemented) and some will even be saying; I want to be tested from that side “PROMISE’. You need to teach us what we are missing” (NGO counsellor; individual interview)

## Implementation

### What combination of implementation effects influenced the outcome results?

#### Innovation

The most appreciated tool of the PROMISE strategy for mothers, PROMISE-EPI staff and HCP was that laboratory tests were available on the same day and therefore clinical decisions could be made immediately: initiation of ART in case of HIV infected infants, adapted ARV adherence counselling and lamivudine initiation in case of unsuppressed maternal viral load. Activities related to the protection of the children could not be initiated in the participants of control group as they had to wait for VL results.

“Most mothers in the control group were not happy because they would not get their viral load results there and then, they would wait until 12 months, by which time they find that it’s very high, and others would have their children test positive for HIV, maybe because they never received their results at 6 months, or at every 3 months that they were being tested at the national programs used to be a challenge.” (PROMISE-EPI staff; FGD)

The two recommendations given by the interviewees to improve PROMISE-EPI intervention were to follow-up the infants up to the end of the at risk period (2 years-old) and to extend the services to partners.

On the other hand, secondary elements of the PROMISE-EPI prevention package from which participants in the intervention and control arm, as well as the HCP, benefited were highlighted by the interviewees. They mentioned the significant impact of these elements; namely: a warm welcome; a dedicated space; detailed and dedicated counselling; reimbursement for transport; solar panels and batteries; reminders and additional staff.

#### Individuals

Many mothers reported that they felt confident thanks to the warm welcome, and non-judgmental listening of the PROMISE-EPI team.

“You never judged me, you showed me love, counseled me and helped me start treatment.” (Mother 27 yrs; FGD)

Some interviewees reported an inappropriate attitudes of some HCPs outside the trial. In PROMISE-EPI, they appreciated the help received when the baby was sick and the prompt drug refill, which was sometimes difficult to achieve outside of the trial. Mothers mentioned that they would prefer getting more doses of study drug because coming back to the health facility frequently can be challenging.

“For me I had a problem at home, at times he (partner) would get the medicine and throw it away, then I would still come back... and because of your understanding, you would give me some more.” (Mother 33 yrs; FGD)

The installation of tents and a container reduced space constraints and therefore the fear of stigma for some mothers. However, having to go to a place dedicated to the study did not make some PROMISE-EPI participants feel comfortable.

“I also never encountered any problem with my child taking this medication, the only challenge I had was, when coming for (drug) collection, I was scared, in case anyone who knows me asks me what I was doing at the container.” (Mother 25 yrs; FGD)

Many appreciated getting reimbursement for transportation and a reminder for their appointment in case they forgot. Furthermore, appointments given in advance allowed some mothers to organize themselves to be available for the next consultation.

#### Inner setting

The PROMISE-EPI study has contributed to good PMTCT outcomes in the health facilities where it has been implemented, by reducing the loss to follow-up. The dedicated staff and communication of laboratory results obtained during the trial have reduced the overload on MCH services and allowed HCP, such as counselors, to do their job more easily.

“The baby- mother pair register was filled in with results from PROMISE and had improved the dashboard performance for Matero clinic.” (counsellor; individual interview)

#### Outer setting

Solar panels and batteries were installed to cover the electricity needs of the GeneXpert machines during frequent power outages. Nevertheless, the charging system for the panels/batteries was not efficient enough to meet the needs of the study in the second year, which was problematic mainly during the rainy season, due to insufficient sunlight. Samples were then sent to University Teaching Hospital to be tested during the day.

The COVID-19 pandemic negatively affected the PROMISE-EPI trial in different ways. As described in REACH, contextual factors related to the COVID-19 pandemic had a negative impact in terms of PROMISE-EPI fidelity. Restrictions and the fear to visit health care facilities increased the number of missed visits and the rate of loss to follow up.

Another consequence of the pandemic was the stock out of GeneXpert cartridges during the trial for a few months. Not having results on the same day of sample collection decreased mothers' confidence in the study, leading to difficulty in counselling and increased loss to follow up.

“Mothers will come at first and we would tell them we will have cartridges the next week then when they come they find no cartridges so it was difficult to even get a blood sample from the mother especially those who were in the intervention arm because they were used to giving the sample and getting a result, but this time we were telling them we were sending them to UTH, so they would ask a lot of questions which showed some doubts” (PROMISE-EPI staff, individual interview)

Furthermore, the study ran out of lamivudine syrup for one month for all participants because of a replenishment delay during the COVID-19 pandemic. It was temporarily replaced by dispersible tablets of abacavir/lamivudine (ABC/3TC) which were more difficult to prepare, according to mothers.

A barrier to remaining in the study was related to community or family distrust of research projects in general. In addition, the use of a different (from guidelines) drug regimen led to rumors from the community.

“I had one challenge with my cousin. When I told her about the PROMISE study, she overreacted and said: you should have been taking your baby to a government clinic for his medication, the study people are just lying to you, your baby will be infected”. (Mother 29 yrs; FGD)

## Maintenance

### What are the facilitators and barriers to maintaining the PROMISE-EPI prevention package?

At the end of the PROMISE-EPI study, the intervention could not be continued. Lamivudine is not approved as single infant PNP and routine laboratory testing is mainly performed in central laboratories. Despite the fact that a GeneXpert machine was available in one of the healthcare facilities, HCP reported that results were not immediately available due to reagents stock out. Proper maintenance of the machine and availability of reagents are necessary to provide optimal services.

“We have a point of care machine but sometimes we run out of cartridges and this has been the major challenge. So, we are always forced to send our DBS samples to another lab where now results take so long to come out. Turnaround time is poor, usually after 2–3 months is when we receive the results.” (Nurse; individual interview)

Furthermore, the close follow-up provided in PROMISE-EPI was possible thanks to dedicated staff. Outside the study, understaffing in health facilities leads to long waiting times and lack of counselling.

“Last time when I was doing observation in MCH under PMTCT, there was only one midwife against over 40 mothers who came to seek MCH services, and that on its own is a very big challenge being faced at the facility.” (PROMISE-EPI staff; individual interview)

## Discussion

This qualitative study helped to identify barriers and facilitators to the PROMISE-EPI trial and specifically the PROMISE-EPI prevention package, to identify quality gaps and generalizable knowledge. The immediate medical action made possible by same-day viral load results was a major benefit identified in the study, with several downstream results such as increased adherence to ARVs and retention in care due to visits perceived to be more beneficial. A randomized controlled trial on point of care compared to standard laboratory viral load testing in South Africa had a similar interpretation of their results [21]. The PROMISE-EPI trial results also support this conclusion, with a higher proportion of loss to follow-up among mothers virally unsuppressed at baseline in the control arm compared with the intervention arm [7]. In addition, if POC for maternal viral load was implemented routinely, healthcare professionals expected their motivation to increase due to their greater scope of immediate action. The WHO has recommended point-of-care viral load testing for pregnant and breastfeeding women since 2021 [5]. Nevertheless, scaling up this technology requires precise organization in settings and country level in terms of supply chain, reagents forecasting, machine maintenance and human resources [22]. In real-world settings, inconsistent supply chain management of laboratory reagents had already been identified as one of the principal factors impeding PMTCT laboratory services [23–25], and was identified as barriers to MAINTENANCE in our study. PROMISE-EPI trial experienced the negative consequences of sub-optimal use of the POC, with 5% of M6 visits for which VL results were not available on the same day. This was due to a lack of reagents in the context of the COVID-19 pandemic, bringing the monitoring of maternal care closer to that of real life. The actual impact of receiving real-time results on mothers’ attendance was broken, with a drop in confidence and a decline in attendance.

Although PROMISE-EPI was a randomized controlled trial, conducted under the best conditions to test the efficacy of the prevention package, its implementation was subject to a major external factor creating an unprecedented context. Indeed, the barriers of PROMISE-EPI were mainly linked to COVID-19 pandemic. In addition to the consequences of the shortage of GeneXpert cartridges described above, REACH was negatively affected by a drop in attendance at health facilities. These findings are consistent with previous qualitative Zambian studies where fear of COVID-19 exposure and COVID-19 prevention measures, such as wearing a mask, were also identified as barriers to care [26, 27].

Stigma hinders continuum of HIV care. Some elements of the PROMISE-EPI trial have contributed overall to reducing stigma and discrimination among participants. Having a dedicated space was a double-edged sword: it made mothers feel more confident once inside, but could cause stress when they entered the room. Comprehensive training and dedicated staff ensured a warm welcome without judgement and or inappropriate attitudes, as happens in real life [28]. It also made it possible to offer substantial counselling that empowered mothers who were less receptive to rumors and facilitated HIV status disclosure with their partners. In a meta-analysis on 14 African countries, partner support was found to be one of the major factors for good maternal adherence to ARVs [29]. This may be explained by the deep-rooted patriarchal societies in Zambia, that hinders womens’ autonomy [30]. However, gender inequalities can also result in a reduction in the well-being of the mothers and their infants in case of status disclosure to the partner [31, 32], which argues in favor of improving gender equity at the same time as involving male partners in PMTCT [30].

The secondary elements of the PROMISE-EPI prevention package, including those described above as helping to reduce stigma, had a positive impact on individuals (PROMISE-EPI participants of both arms) and on inner settings (MCHs). POC offered for infant HIV diagnosis at EPI-2, M6 and M12 in both arms was also considered a facilitator. These results suggest that the participants in the control group were better followed than in the real life as a result of ‘study effect’, thereby reducing the rate of loss to follow up and MTCT. The secondary elements may also have had an effect on the behavior of the virally unsuppressed PROMISE-EPI mothers in the intervention arm compared with those in the control arm. Indeed, this subgroup of mothers in the intervention arm, proportionally better retained than those in the control arm according to the results of the main trial [7], were more likely to benefit from the secondary elements because of the monthly visits for study drug refills. Being able to measure the weight of the secondary elements in the results of the PROMISE-EPI trial would make possible assessing the gap between the evidence-based practice of PROMISE-EPI prevention package (POC and single PNP) in research and in real life settings. A new implementation study, called PROMISE-ZERO, will evaluate only the impact of the PROMISE-EPI prevention package in urban and rural areas of Zambia’s Eastern province, and in Lusaka.

Limitations of our study were mainly related to possibility of social desirability bias. PROMISE-EPI staff, after appropriate training, were considered the most suitable for interviewing. Indeed, the interviewers were able to ask the questions given their knowledge of the context, the mothers had trust in them and they were more confident in expressing themselves freely with the health care professionals who had been following them for several months. However, this may have induced a social desirability bias. This bias may have occurred during the interviews with the PROMISE-EPI staff themselves. Nevertheless, health care professionals not involved in PROMISE-EPI also contributed greatly to the analysis on ADOPTION with a convergent and complementary vision. Another limitation is that the interview guide did not include specific questions on the consequences of the challenges faced during COVID-19 (lamivudine and cartridges stock out). Finally, the interviewed PROMISE-EPI participants were more likely to have appreciated the intervention offered in PROMISE-EPI, as shown by the difference in the loss to follow up rate for the PROMISE-EPI M12 visit between mothers who were interviewed and those who were not.

## Conclusion

This qualitative study provides new elements to better understand the reduced HIV transmission with the PROMISE-EPI intervention. It also highlights potential gaps between the package proposed in the trial and what can be applied in less controlled, ‘real life’ settings, namely the secondary elements of the PROMISE-EPI package that will be investigated in depth in the PROMISE-ZERO implementation study.

## Supplementary Information

Supplementary Material 1.

Supplementary Material 2.

## Data Availability

The datasets used and/or analysed during the current study are available from the corresponding author on reasonable request.

## Abbreviations

ART: Antiretroviral therapy
ARV: Antiretroviral
CFIR: Consolidated Framework for Implementation Research
EPI-2: Second visit of the Expanded Program on Immunization
FGD: Focus Group Discussions
HCP: Health Care Professional
MCH: Maternal and Child Health
MTCT: Mother To Child Transmission
PMTCT: Prevention of Mother to Child Transmission
PNP: Post-Natal Prophylaxis
POC: Point-Of-Care
RE-AIM: Reach, Effectiveness, Adoption, Implementation, Maintenance
VL: Viral Load
