# Supplementary material for: Barriers and facilitators of a large clinical trial on prevention of HIV transmission through breastfeeding in Lusaka, Zambia: a qualitative study
Source: BMC Public Health. 2024 Dec 2;24:3356. doi: 10.1186/s12889-024-20855-5 (PMC11613839; doi:10.1186/s12889-024-20855-5)
Supplement: Supplementary file 1 — Supplementary Material 1. [file 12889_2024_20855_MOESM1_ESM.docx]

**Appendix**

Table 1: Study findings split between the 5 CFIR domains in the five RE-AIM components

| **RE-AIM** | **CFIR domains** | **B/F** | **Description** |
| --- | --- | --- | --- |
| **REACH** |  |  |  |
| INDIVIDUALS | | B* | Maternal obligations and challenges : caring for other children, financial challenges and travel for personal reasons or to deliver near family |
|  |  | B* | Denial, self-stigmatization |
|  |  | B* | Facility mistrust |
| INNER SETTING | | B* | Age gap between mothers |
|  |  | B* | Inadequate information received |
| OUTER SETTING | | B | COVID19: barriers to EPI-2 attendance |
|  |  | B* | Cultural practices |
| **EFFECTIVENESS** | | | |
|  | INDIVIDUALS | F | Decrease mothers' anxiety, useful visit: increase trust in health care system and improve attendance |
|  |  | F | Mothers’ fear of being reprimanded, concrete impact of ARVs intake: improve ARVs adherence |
|  |  | F | More confident mothers, empowered mothers: less receptive to rumors, facilitate HIV status disclosure; breastfeeding longer |
| **ADOPTION** | | | |
|  | INDIVIDUALS | F | New skills (GeneXpert machine) |
|  |  | F | Immediate and appropriate care given by the caregivers: increase HCPs motivation |
| INNER SETTING | | F | HCPs’ willingness to learn more about PROMISE-EPI intervention and trial results |
| **IMPLEMENTATION** | | | |
| INNOVATION | | F | POC: Immediate medical decisions possible (counselling; ART; lamivudine PNP) |
|  |  | B | Recommendation of children follow up until 2 years old |
|  |  | B | Recommendation to extend services to partners |
|  |  | NA | Secondary elements: warm welcome; dedicated space; transport refund; reminders; additional staff; substantial counselling; solar panels and batteries |
|  |  | B | Element related to the research: frequent visits; unusual drug |
|  | INDIVIDUALS | F | Warm welcome without judgement and help received : confident mothers |
|  |  | B | Challenges of frequent visits |
|  |  | F | Space issue decreased: fear of stigma decreased |
|  |  | B | Dedicated space: fear of stigma |
|  |  | F | Transport refund : decrease of financial burden |
|  |  | F | Reminders : improved retention |
|  | INNER SETTING | F | Health facility workload reduced thanks to PROMISE-EPI and retention increased |
|  |  | F | Good communication between health facility and study staff |
|  | OUTER SETTING | B | Power outage: need to install solar panels and batteries |
|  |  | B | Space issue: need to provide dedicated space |
|  |  | B | COVID19: barrier to attendance; stock out of GenXpert cartridges and lamivudine |
|  |  | B | Community burden: mistrust of research and rumors about unusual drugs |
| **MAINTENANCE** | | | |
|  | INNOVATION | B | Intervention externally developed, not maintained at the end |
|  | INNER SETTING | B | Health facility understaffed |
|  |  | B | GeneXpert and/or cartridges not available |

*B: Barriers; F: Facilitators; NA: Not applicable; ARV: Antiretrovirals; ART: Antiretroviral therapy; EPI-2: Expanded program on immunization-second visit; HCP: Health care professionals; PNP; Post-natal prophylaxis; POC: Point-of-care;*

** Not specifically reported for EPI-2, but for MCH attendance*
