# Supplementary material for: Barriers and facilitators of a large clinical trial on prevention of HIV transmission through breastfeeding in Lusaka, Zambia: a qualitative study
Source: BMC Public Health. 2024 Dec 2;24:3356. doi: 10.1186/s12889-024-20855-5 (PMC11613839; doi:10.1186/s12889-024-20855-5)
Supplement: Supplementary file 2 — Supplementary Material 2. [file 12889_2024_20855_MOESM2_ESM.pdf]

UTH and INSERM
**EXPLORATION OF FACTORS INFLUENCING THE PREVENTION OF MOTHER-TO-CHILD TRANSMISSION OF HIV-1 PROGRAMS EFFECTIVENESS IN ZAMBIA**<br><br>**FOCUS GROUP DISCUSSION (FGD) AND INTERVIEW GUIDE**

The objectives of the project:

- **To explore the factors influencing the effectiveness of the existing PMTCT program follow up**
  - To explore contextual challenges (including healthcare organization); and individual factors influencing mother's adherence to the PMTCT program
- **To explore the factors influencing the effectiveness of PROMISE-EPI intervention**
  - To explore the factors related to mothers' and healthcare professional acceptability, feasibility and fidelity to the PROMISE-EPI intervention
- **To explore the factors associated with the acceptability, feasibility and usability of Zambian recommendations compared to PROMISE-EPI intervention**
  - To explore factors related to the acceptability of a triple-drug prophylaxis versus single-drug PrEP (lamivudine) and quarterly viral load tests using central lab versus bi-annual Point of Care viral load
  - To explore the contextual factors influencing the effectiveness of Zambian policy

1. **INTRODUCTIONS**

WELCOME remark. Thanks for agreeing to be part of the focus group. We appreciate your willingness to participate.

INTRODUCTION of the***: Moderator/Assistant Moderator/others :***
My name is _____________________________________and I work with the University Teaching Hospital. We are interested in documenting your experiences, benefits and challenges while in the PROMISE-EPI study.

2. **CONFIDENTIALITY AND CONSENT:**

You have been selected to take part in group discussions. You provided consent to take part in this group discussion when you signed on the consent form for participation in the study. However, you are free to opt out of the group discussion at any time.

3. **SELECTION OF PARTICIPANTS**

You have been selected based of the data recorded for the main study.

4. **FOR THE GROUP DISCUSSION**
Each group will consist of 6-10 participants and the groups have been chosen based on the following criteria.
   - **Mothers who dropped out of the PMTCT cascade (3 FGDs)**
     - HIV positive mothers whose child had not been screened for HIV immediately post-delivery or whose child did not receive ART prophylaxis at birth.
5. **FOR THE INTERVIEWS**
   - **HIV positive mothers whose child received the two types of prophylaxis** (lamivudine/3-drug prophylaxis) (8 interviews)
   - **HIV positive mothers unsuppressed** at EPI-2 and still unsuppressed at M6 and M12 or suppressed at EPI-2 and unsuppressed at M6 (all mothers who fit criteria at time of study)
   - **HIV positive mothers still breastfeeding at 12 months** (10 interviews)
   - **Health care providers** (15 interviews)

CONDUCT OF THE GROUP DISCUSSIONS: *The discussion will be led by a study staff member with the assistance of another who will be writing down important information. The discussion will may take about 1 and a half hours of your time. Each group will come up with group norms which every member must abide by. Before we start the discussion, we want to cover general guidelines for the discussion so we can be sure that each of you has a chance to talk as we share ideas.*

6. **GROUND RULES**

WE WANT YOU TO DO THE TALKING.
   a. *I would like everyone to participate. I am simply interested in understanding your opinions and thoughts. You are not required to answer any of the questions if you do*

not feel comfortable doing so. I need your input and want you to share your honest and open thoughts with us.

b. *I may call on you if I haven't heard from you in a while.*

THERE ARE NO RIGHT OR WRONG ANSWERS

a. *Every person's experiences and opinions are important. Speak up whether you agree or disagree. We want to hear a wide range of opinions.*

WHAT IS SAID IN THIS ROOM STAYS HERE

b. *We want you to feel comfortable sharing when sensitive issues come up.*

WE WILL BE TAPE RECORDING THE GROUP

c. *We want to capture everything you have to say. We don't identify anyone by name in our report. You will remain anonymous.*

DURING OUR DISCUSSION, WE SHALL FOLLOW THE ADDITIONAL GUIDELINES BELOW.

- *Each one of you will use either a true or false name depending on your choice.*
- *We will accept one response at a time; please be sure to let the previous person finish before you give your answer*
- *and respect their opinions.*
- *Please speak loudly in order for everyone to hear what you are saying.*
- *There is no wrong response; all responses are important. You are free not to respond to any question.*

7. DO YOU HAVE ANY QUESTIONS BEFORE WE START THE DISCUSSION?

[*FACILITATOR STARTS THE TAPE RECORDER AND READS:*]

This discussion will be tape recorded. Before we start, please verbally confirm that you are aware that we are tape recording this session and that it is okay with you *[please get a verbal okay from all members of the focus group before continuing].*

SECTION A: To explore the contextual (COVID-19, rainy season, local and nationwide stockouts, other) and individual factors influencing PMTCT program follow-up.

A1) Mothers who dropped out of the PMTCT cascade: HIV positive mothers whose child had not been screened for HIV immediately post-delivery (samples not taken or results not received) or whose child did not receive ART prophylaxis at birth. (4 FGDs)

We understand that within this group are mothers who have experienced challenges in finding or receiving care for your children for prevention of HIV infection. Some of you may not know the HIV status of your children: some may not have screened for HIV, some of you may not have received the HIV results for your children; some of your children may be HIV positive but may not be receiving HIV treatment:

1. *Share with us, which one of these is your experience/challenge? (No PCR for your baby a birth or no PCR result received on time/ no PrEP given to the child at birth or PrEP for less than 6 weeks)*

2. *A) What are some of the problems that you encountered that led to these challenges? (Probes that could influence the drop out of the PMTCT cascade)*

   <u>Community or wider issues</u>:
   - *Climate issues (rainy season, etc.)*
   - *COVID and associated restrictions,*
   - *Cultural factors (black magic, churches, fake news…)*

   <u>Health facility level</u>
   - *Quality of care, trust in health care providers*
   - *Drug stock outs*
   - *Long turnaround time for screening and results, long queues*
   - *Loss of ART cards*

   <u>Aspects of personal lives (please describe)</u>
   - *Lack of food, transport money*
   - *Aspects of drugs and requirements; including forgetting to take medication*
   - *Aspects of social support structures (stigma), family support, spouse support; non-disclosure to spouse*
   - *Work commitment*

B) Do you have any challenges with your medical care follow-up? Do you know how often you should perform a test on your viral load (out of PROMISE-EPI)? Do you have any recall? How long is it to get the result?

What else?

3. *What are some ways you can suggest to improve these services and allow more women and their children access these services?*

4. *In your opinion, how have you benefited from being part of the study?*

5. *Wrap up.*
    - *We've now reached the end of our discussion. Do you have any questions or any additional comments?*
    - *Thank you for taking the time to talk to me today and share your opinions. We truly appreciate your willingness to participate and discuss your experiences with us.*
    - ***At this time, the facilitator should answer any unanswered questions and clarify misconceptions, or refer participants to someone who can.***
    - ***Ensure proper documentation.***

A2) HIV negatives mothers whom Prep was proposed during pregnancy: PrEP proposed to mother during pregnancy but not accepted/Prep taken during pregnancy but stopped before EPI-2/ Prep continued after EPI-2 (2 FGD+ 1 individual interview)

We would like to hear about your experience on PrEP:

1-Why did you decide to take the Prep/ to not take Prep during your pregnancy and during breastfeeding?
2-Have you experienced any challenges in taking PrEP? (Lack of information on how to take PrEP, difficulty in getting a refill, family or community issues…)
3- When did you decide to stop PrEP and why?
4- Did you have creatinine clearance and ALT (term to be explained to mothers) checked before PrEP initiation and during PrEP use

A3) HIV positive mothers whose baby was diagnosed in PROMISE-EPI (at EPI-2) (3 Focus group)

We would like to hear about your experience on the medical care your child received since his HIV diagnosis

1- When were you diagnosed for HIV? Did you take ARV during your pregnancy? Did you have the viral load checked during pregnancy? What was the result? Did you disclose your status with your partner?

2-Are you satisfied with the medical care received by the ART clinic after your child diagnosis? Did the health care providers answer all your questions? Did you have access to counselling sessions? If no, why? If yes, was it useful?
3- Do you feel comfortable with the ARV to be given to your child? Do you experience any challenges? (lack of information on how to take PrEP, difficulty in getting a refill, family or community issues…)
4- When was the last viral load test for your child? How long did you wait for the result? What was the result? Were some actions taken following the result (counselling sessions, ARV treatment modification: 2nd line…)?

SECTION B (I): Health care providers and key stakeholders (5 interviews + 7 interviews of the PROMISE-EPI staff + 1 FGD of the PROMISE-EPI staff)

a) There are three main questions in this section of the interview.

1) the contextual factors influencing PMTCT program efficiency

2) the factors related to the acceptability, feasibility and fidelity of the intervention used in PROMISE-EPI study

3) Comparison of factors associated with the acceptability, feasibility, adherence and usability of Zambian recommendations to those of the PROMISE-EPI intervention

You have been invited to take part in this interview because you are a health care provider that work closely with women seeking mother to child transmission (MTCT) of HIIV interventions services. You are familiar with the national recommendations for Zambia for the P*reventions* ***of M****other* ***T****o* ***C****hild* ***T****ransmission* ***(PMTCT) of HIV. Some of you have or are participating in the PROMISE-EPI study and are familiar with the PMTCT interventions being proposed in the research.***

1. *Please share with us some of these recommendations in the Zambian PMTCT guidelines and in the PROMISE-EPI research study?*

2. *Please describe what work you do in the PMTCT program?(list down the cadres of health workers)*

3. *Describe some of the challenges experienced by your facility and by the mothers and their children that challenge access to PMTCT services. What are some of these challenges you have experienced or noticed in your work?*

   Possible probes:
   1) The contextual factors influencing PMTCT program efficiency
   - Are there specific sub-groups not able to attend or less likely to attend follow up?

- What reasons might these groups be more likely/less likely to attend PMTCT and EPI2 visits?
    - To not receive Child HIV PCR results at EPI-2 (samples taken at birth);
    - To be less adherent to ARV (mother) or to PrEP (less than 6 weeks PrEP for infant);
    - To not follow the national program in case of unsuppressed viral load (monthly counselling sessions for 3 months followed by a new viral load test and switch to 2nd line treatment in case of still unsuppressed)
    - Aspects of participants' personal lives
        - (i.e. work commitments, fear or lack of support from partners, etc.)
        - Disclosure to significant others
        - Economic challenges
    - Aspects of the community/the place where the participants live
        - (i.e. transport, safety, distrust of research, rumors, violence, climate issues, COVID and associated restrictions, HIV Stigma etc.)
        - Personal or community support structures
    - Aspects at facility level
        - Local and national availability of ARV drugs/stock outs of reagents or drugs
        - Turnaround time for test results
        - Personnel and equipment capacities
        - Community attitudes and acceptability of the program

2) The factors related to the acceptability, feasibility, adherence, and usability of the intervention used in PROMISE-EPI study:

- Requirements of taking lamivudine PrEP for infants like formulation, schedule of administration.
    - Aspects of participants' personal lives
        - (i.e. work commitments, fear or lack of support from partners, etc.)
        - Disclosure to significant others
    - Aspects of the community/the place where the participants live
        - (i.e. transport, safety, distrust of research, rumors, violence, climate issues, COVID and associated restrictions, HIV Stigma etc.)
        - Personal or community support structures
    - Aspects at facility level
- Challenges faced when using the Point of Care test and lessons learned from PROMISE-EPI study

3) Comparison of factors associated with the acceptability and feasibility of Zambian recommendations to those of the PROMISE-EPI intervention

- *Factors related to the acceptability of a triple-drug prophylaxis versus single-drug PrEP (lamivudine)*
    - *Aspects of participants' personal lives*
    - *Aspects of the community/the place where the participants live*
    - *Aspects at facility level (Local and national availability of ARV drugs)*
- *Factor related to the acceptability and effectiveness of a quarterly viral load tests using central lab versus bi-annual Point of Care viral load*
    - *Aspects of participants' personal lives*
    - *Aspects of the community/the place where the participants live*
    - *Aspects at facility level*
        - *Local and national stock outs of reagents*
        - *Turnaround time for test results*
        - *Personnel and equipment capacities to carry out new vs old Zambian guidelines*
        - *Community attitudes and acceptability of the program*

4. **What are some ways you can suggest to improve these services and allow more women and their children access the PMTCT services?**

5. **In your opinion, how have you benefited from being part of the study?**

6. **Wrap up***.*
    - *We've now reached the end of our discussion. Do you have any questions or any additional comments?*
    - *Thank you for taking the time to talk to me today and share your opinions. We truly appreciate your willingness to participate and discuss your experiences with us.*
    - ***At this time, the facilitator should answer any unanswered questions and clarify misconceptions, or refer participants to someone who can.***
    - ***Ensure proper documentation.***

SECTION B(II): Key stakeholders/Managers of the PMTCT programs (5 interviews)

The main question in this section of the interview: is to elicit the contextual factors influencing PMTCT program efficiency

You have been invited to take part in this interview because you are a health care provider and manager that work closely with women seeking mother to child transmission (MTCT) of HIIV interventions services. You are familiar with the national recommendations for Zambia for the P**reventions **of M**other **t**o **C**hild **T**ransmission **(PMTCT) of HIV. Some of you have or are participating in the PROMISE-EPI study and are familiar with the PMTCT interventions being proposed in the research.

4. *Please share with us what work you do in the PMTCT program?*
5. *Please share with us your statistics of the PMTCT cascade at your institution?*
6. *Please describe the human resource capacity at your PMTCT unit? And what capacity would you consider adequate to execute the work required in the unit?*
7. *Describe any challenges with testing for HIV and viral load of women and their children? What is the turn round time for these tests at your institution?*
8. *Describe the challenge with stockouts at your institution? laboratory reagents? rapid test kits? and HIV drugs?*
9. *Please describe the training provided to the MCH unit staff in the national PMTCT guidelines? Any training conducted this year? Job aide available to your staff in the MCH unit? Share with us what job aide you currently have?*
10. *How do your staff use the available guidelines? What are the challenges? Probe on availability?*
11. *Please describe which part of the PMTCT cascade most of your patients fall out? Do you know the reasons why?*
12.
    a. *How has the COVID-19 pandemic affected the PMTCT services your institution is offering?*
    b. *How has the PROMISE-EPI study and its recommendations affected the PMTCT services your institution is offering?*
    c. *What are some ways you can suggest to improve these services so that more health workers and more institutions are equipped to provide a better PMTCT service?*
13.
    a. *How has the COVID-19 pandemic affected the people around this community that seek PMTCT services?*
    b. *How has the PROMISE-EPI study and its recommendations affected the people around this community that seek PMTCT services?*
    c. *What are some ways you can suggest to improve these services and allow more women and their children access the PMTCT services?*
14. *In your opinion, how have you benefited from being part of this study?*
15. **Wrap up**.
    - *We've now reached the end of our discussion. Do you have any questions or any additional comments?*

- *Thank you for taking the time to talk to me today and share your opinions. We truly appreciate your willingness to participate and discuss your experiences with us.*

- ***At this time, the facilitator should answer any unanswered questions and clarify misconceptions, or refer participants to someone who can.***

- ***Ensure proper documentation.***

SECTION C: HIV positive mothers whose child received the two types of prophylaxis (lamivudine/3-drug prophylaxis) (4 interviews + 4 FGDs)

You have been invited to take part in this interview because you are a mother that has accessed ***P****reventions* ***of M****other* ***T****o* ***C****hild* ***T****ransmission* ***(PMTCT) of HIV services. Some of you have or are participating in the PROMISE-EPI study and are familiar with the PMTCT interventions being proposed in the research.***

1. ***Exploration of factors relating to the effectiveness of the existing PMTCT program follow up***

   a. ***Please, describe what your experience has been with the PMTCT services?***

   Probes: When did register for PMTCT, what was the age of the pregnancy or child? When was your child tested for the last time (was it within PROMISE-EPI)? What were the results? had your child been tested for HIV out of PROMISE-EPI? At birth? When did you get the results? When was prophylaxis started (tri-therapy and lamivudine)? What PrEP is the child on, if any?

   b. **What can lead HIV positive mothers to have difficulty adhering to ARV treatment? Do you sometimes miss taking your ARV medication? If yes, why?**
      - Feasibility factors including health facility factors; personal, relational that make it unfeasible to adhere to ARV treatment
      - Acceptability factors including reasons that the ARV regimens were not acceptable to participants including personal, cultural, relational factors
      - Fidelity factors; these are factors that show that the program or the study were not implemented according to the guidelines (adherence counselling session; Viral load test, ARV switch to 2nd line treatment)

   c. **Exploration of factors related to lamivudine PrEP adherence in newborns**
      - What reasons might you be more likely/less likely to adhere?

- Drug side effects
- Requirements of taking lamivudine like formulation, schedule of administration
- Aspects of participants' personal lives
    - (i.e. work commitments, fear or lack of support from partners, etc.)
    - Disclosure to significant others
    - Economic challenges
- Aspects of the community/the place where the participants live
    - (i.e. transport, safety, rumors, violence, HIV Stigma)
    - Personal or community support structures
- Aspects at facility level
    - Personnel and equipment capacities within the study
    - Community attitudes and acceptability
- Please discuss how providing medication for your child is different to taking medication for yourself. Is it harder or easier? Why, why not?

2. **Exploration of factors related to the acceptability of triple-drug prophylaxis versus single-drug PrEP (lamivudine)**
    - Please discuss how you feel about the difference in the 3 drugs vs single drug regimen
        - Anything about timing? Swallowing? Other?
        - Family or community issues
        - Aspects at facility level: difference in care received within the study (lamivudine provided) / out of the study (3 drug prophylaxis provided) (for example: enough information given on how to take the drug? Difficulty in getting a refill?)

3. **Exploration of factors related to the acceptability of quarterly viral load by central lab compared to bi-annual viral load (including one when the infant is 6-8 weeks) using Point of Care.**
    - Do you know how often you perform a test on your viral load (out of PROMISE-EPI)? Do you have any recall?
    - Please discuss your experience with the quarterly vs bi-annual viral load (including one when the infant is 6-8 weeks) using Point of Care.
        - Anything about transport, time, other?
        - Aspects of the care received at facility level: difference in care received within the study (point of care) / out of the study (central lab)
4. **What are some ways you can suggest to improve these services and allow more women and their children access the PMTCT services?**

5. **In your opinion, how have you benefited from being part of the study?**

6. **Wrap up***.*

- *We've now reached the end of our discussion. Do you have any questions or any additional comments?*

- *Thank you for taking the time to talk to me today and share your opinions. We truly appreciate your willingness to participate and discuss your experiences with us.*

- ***At this time, the facilitator should answer any unanswered questions and clarify misconceptions, or refer participants to someone who can.***

- ***Ensure proper documentation.***

We've now reached the end of our discussion. Do you have any questions or any additional comments?

SECTION D: HIV positive mothers unsuppressed at EPI-2, M6 and M12 or suppressed at EPI-2 and unsuppressed at M6 or suppressed at EPI-2 and M6 and unsuppressed at M12 (7 interviews)

You have been invited to take part in this interview because you are a mother that has accessed ***P****reventions* ***of M****other* ***T****o* ***C****hild* ***T****ransmission* ***(PMTCT) of HIV services. Some of you have or are participating in the PROMISE-EPI study and are familiar with the PMTCT interventions being proposed in the research. You are also at different stages of receiving interventions of PMTCT.***

1. ***Exploration of factors relating to the effectiveness of the existing PMTCT program follow up***
    a. **What has your experience been with the PMTCT services?**
    Probes: When did register for PMTCT, what was the age of the pregnancy or child? When was your child tested for the last time (was it within PROMISE-EPI)? What were the results? Had your child been tested for HIV out of PROMISE-EPI? When did you get the results? When was prophylaxis started (triple prophylaxis and lamivudine)? What PrEP is the child on, if any?

    b. **We would like to further hear from you what you think or what your experience has been with the following:**

        i. Do you have any challenges with your medical care follow-up?

        ii. What can lead HIV positive mothers to have difficulty adhering to ARV treatment? Do you sometimes miss taking your ARV medication? If yes, why?

i. Feasibility factors including health facility factors; personal, relational that make it unfeasible to adhere to ARV treatment

ii. Acceptability factors including reasons that the ARV regimens were not acceptable to participants including personal, cultural, relational factors

iii. Fidelity factors; these are factors that show that the program or the study were not implemented according to the guidelines (adherence counselling session; Viral load test, ARV switch to 2nd line treatment)

c. What can lead to HIV positive mothers not adhere to national program in case of uncontrolled viral load (counselling sessions, new viral load test and finally switched to 2nd line treatment)

i. Feasibility factors including health facility factors; personal, relational that make it unfeasible to adhere to national program

ii. Acceptability factors including reasons that the national program was not acceptable to participants including personal, cultural, relational factors

iii. Fidelity factors; these are factors that show that the national program was not implemented according to the guidelines (adherence counselling session; Viral load test, ARV switch to 2nd line treatment)

2. Exploration of factors related to lamivudine PrEP adherence in newborns

- What reasons might you be more likely/less likely to adhere?
  - Drug side effects
  - Requirements of taking lamivudine like formulation, schedule of administration
  - Aspects of participants' personal lives
    - (i.e. work commitments, fear or lack of support from partners, etc.)
    - Disclosure to significant others
    - Economic challenges
  - Aspects of the community/the place where the participants live
    - (i.e. transport, safety, rumors, violence, HIV Stigma)
    - Personal or community support structures
  - Aspects at facility level
    - Personnel and equipment capacities within the study
    - Community attitudes and acceptability
  - Please discuss how providing medication for your child is different to taking medication for yourself. Is it harder or easier? Why, why not?

3. **Exploration of factors related to the acceptability of triple-drug prophylaxis versus single-drug PrEP (lamivudine)**
    - Please discuss how you feel about the difference in the 3 drugs vs single drug regimen
        - Anything about timing? Swallowing? Other?
        - Family or community issues
        - Aspects at facility level: difference in care received within the study (lamivudine provided) / out of the study (3 drug prophylaxis provided) (for example: enough information given on how to take the drug? Difficulty in getting a refill?)

4. **Exploration of factors related to the acceptability of quarterly viral load by central lab compared to bi-annual viral load (including one when the infant is 6-8 weeks) using Point of Care.**
    - Do you know how often you should perform a test on your viral load (out of PROMISE-EPI)? Do you have any recall? How long is it to get the result?
    - Please discuss your experience with the quarterly vs bi-annual viral load (including one when the infant is 6-8 weeks) using Point of Care.
        - Anything about transport, time, other?
        - Aspects of the care received at facility level: difference in care received within the study (point of care) / out of the study (central lab)

SECTION E: HIV positive mothers still breastfeeding at 12 months (6 interviews + 3 FGDs at each study site)

1. ***Exploration of factors relating to the effectiveness of the existing PMTCT program follow up***
    a. **What has your experience been with the PMTCT services?**
    Probes: When did register for PMTCT, what was the age of the pregnancy or child? When was your child tested for the last time (was it within PROMISE-EPI)? What were the results? Had your child been tested for HIV out of PROMISE-EPI? When did you get the results? When was prophylaxis started (triple prophylaxis and lamivudine)? What PrEP is the child on, if any?

    b. **What can lead to women deciding to breastfeed beyond the international recommendations (12 months)**

    Probe on both breastfeeding for all issues:
    i. Cultural factors
        i. What are the cultural beliefs around breast-feeding? Do family members have an influence?

ii. Personal factors
   i. Financial
   ii. Nutritional
   iii. Personal beliefs
   iv. Convenience
   v. Other family members they live with in the house
iii. Community factors
   i. Stigma around stopping breastfeeding?
   ii. **Breastfeeding in public**

2. **Wrap up***.*

- *We've now reached the end of our discussion. Do you have any questions or any additional comments?*

- *Thank you for taking the time to talk to me today and share your opinions. We truly appreciate your willingness to participate and discuss your experiences with us.*

- ***At this time, the facilitator should answer any unanswered questions and clarify misconceptions, or refer participants to someone who can.***

- ***Ensure proper documentation.***

****
